# Label-free Evaluation of Hepatic Microvesicular Steatosis with Multimodal Coherent Anti-Stokes Raman Scattering Microscopy

**DOI:** 10.1371/journal.pone.0051092

**Published:** 2012-11-30

**Authors:** Thuc T. Le, Amy Ziemba, Yasuyo Urasaki, Steven Brotman, Giuseppe Pizzorno

**Affiliations:** 1 Desert Research Institute, Las Vegas, Nevada, United States of America; 2 Nevada Cancer Institute, One Breakthrough Way, Las Vegas, Nevada, United States of America; Beckman Research Institute of City of Hope, United States of America

## Abstract

Hepatic microvesicular steatosis is a hallmark of drug-induced hepatotoxicity and early-stage fatty liver disease. Current histopathology techniques are inadequate for the clinical evaluation of hepatic microvesicular steatosis. In this paper, we explore the use of multimodal coherent anti-Stokes Raman scattering (CARS) microscopy for the detection and characterization of hepatic microvesicular steatosis. We show that CARS microscopy is more sensitive than Oil Red O histology for the detection of microvesicular steatosis. Computer-assisted analysis of liver lipid level based on CARS signal intensity is consistent with triglyceride measurement using a standard biochemical assay. Most importantly, in a single measurement procedure on unprocessed and unstained liver tissues, multimodal CARS imaging provides a wealth of critical information including the detection of microvesicular steatosis and quantitation of liver lipid content, number and size of lipid droplets, and lipid unsaturation and packing order of lipid droplets. Such information can only be assessed by multiple different methods on processed and stained liver tissues or tissue extracts using current standard analytical techniques. Multimodal CARS microscopy also permits label-free identification of lipid-rich non-parenchymal cells. In addition, label-free and non-perturbative CARS imaging allow rapid screening of mitochondrial toxins-induced microvesicular steatosis in primary hepatocyte cultures. With its sensitivity and versatility, multimodal CARS microscopy should be a powerful tool for the clinical evaluation of hepatic microvesicular steatosis.

## Introduction

Hepatic steatosis, or fatty liver, is the earliest stage of non-alcoholic fatty liver disease (NAFLD) commonly associated with metabolic syndrome, drug-induced liver injury, and aging [1]. Hepatic steatosis can be self-contained or can progress into advanced NAFLD stages such as non-alcoholic steatohepatitis (NASH), cirrhosis, and liver cancer [2]. Although NAFLD pathogenesis remains unclear, hepatic steatosis constitutes the first hit and hepatic inflammation constitutes the second hit according to the “two-hit” hypothesis for NASH development [3]. Hepatic steatosis generally develops when the rate of fatty acid input, such as uptake and *de novo* synthesis, exceeds the rate of fatty acid output, such as β-oxidation and export [4]. Conditions that perturb the rates of fatty acid input and output including impaired *de novo* fatty acid synthesis and impaired fatty acid β-oxidation are likely contributors to the development of hepatic steatosis [4]–[6]. Whereas factors that promote oxidative stress and expression of inflammatory cytokines are likely contributors to the progression from hepatic steatosis to NASH [6]. Fatty liver is a significant public health threat in the US due to the obesity epidemic in children and young adults [7], [8], the growing population of elderly [9], [10], and the widespread use of prescription drugs [11], [12].

The gold standard for the diagnosis of hepatic steatosis is histopathology evaluation of liver biopsies. Generally, hepatic steatosis is defined as triglyceride content exceeding 5% of the liver volume or weight [13] or when 5% or more of hepatocytes exhibit visible intracellular lipid droplets [14]. Using histopathology evaluation, hepatic steatosis is qualitatively classified into two forms: microvesicular steatosis and macrovesicular steatosis [14]. Microvesicular steatosis describes cytoplasmic accumulation of small lipid droplets that do not physically perturb the central location of the nucleus. In contrast, macrovesicular steatosis describes cytoplasmic accumulation of large lipid droplets that displace the nucleus from its central location into the cell periphery. However, the staining methods currently used for the evaluation of hepatic steatosis are prone to errors [15]. In hematoxylin and eosin (H & E) stained tissue sections, lipid droplets are evaluated as unstained vacuole regions. While acceptable for macrovesicular steatosis evaluation, H & E staining generally fails to identify microvesicular steatosis [16]. On the other hand, lipid-specific stains such as Oil Red O (ORO) and Sudan IV stain more than just lipid droplets, leading to over-estimation of hepatic steatosis [17], [18] (**Figure S1**). In addition, de-paraffination in xylene prior to staining, a common tissue processing procedure, often leads to loss of tissue lipid content and underestimation of steatosis [16], [18]. Clearly, new methods of detection are needed to improve the sensitivity and accuracy for clinical diagnosis of hepatic steatosis [19].

In recent years, coherent anti-Stokes Raman scattering (CARS) microscopy has been applied to visualize hepatic macrovesicular steatosis in rodents [20]–[22]. CARS microscopy is a label-free imaging technique whose contrast mechanism arises from the intrinsic molecular vibration of the probed samples [23]. CARS microscopy is highly sensitive to the visualization of lipid-rich structures due to the abundance of carbon-hydrogen vibration around 2845 cm^−1^ of the lipid chain [24]. In addition to visualization of hepatic steatosis, CARS microscopy also provides quantitative analysis of lipid content in intact liver tissues that correlates well with biochemical measurement of total lipid extracts [20]. Integrated CARS and second harmonic generation (SHG) imaging permit visualization of steatosis together with fibrosis [21]. Integrated CARS and spontaneous Raman microspectrometry permits analysis of lipid droplet composition [20], [25]. CARS microscopy is emerging as a new and promising technique for the detection of hepatic steatosis and the studies of lipid droplet biology [26].

In this paper, we explore the integrated capability of CARS microscopy for label-free evaluation of hepatic microvesicular steatosis. Analysis data obtained with CARS microscopy on unprocessed and unstained liver tissues are compared with those obtained with histopathology and conventional analytical methods on processed and stained tissues or on total lipid extracts. We aim to evaluate the potential use of CARS microscopy for the clinical diagnosis of hepatic steatosis.

## Results

**Figure 1:**
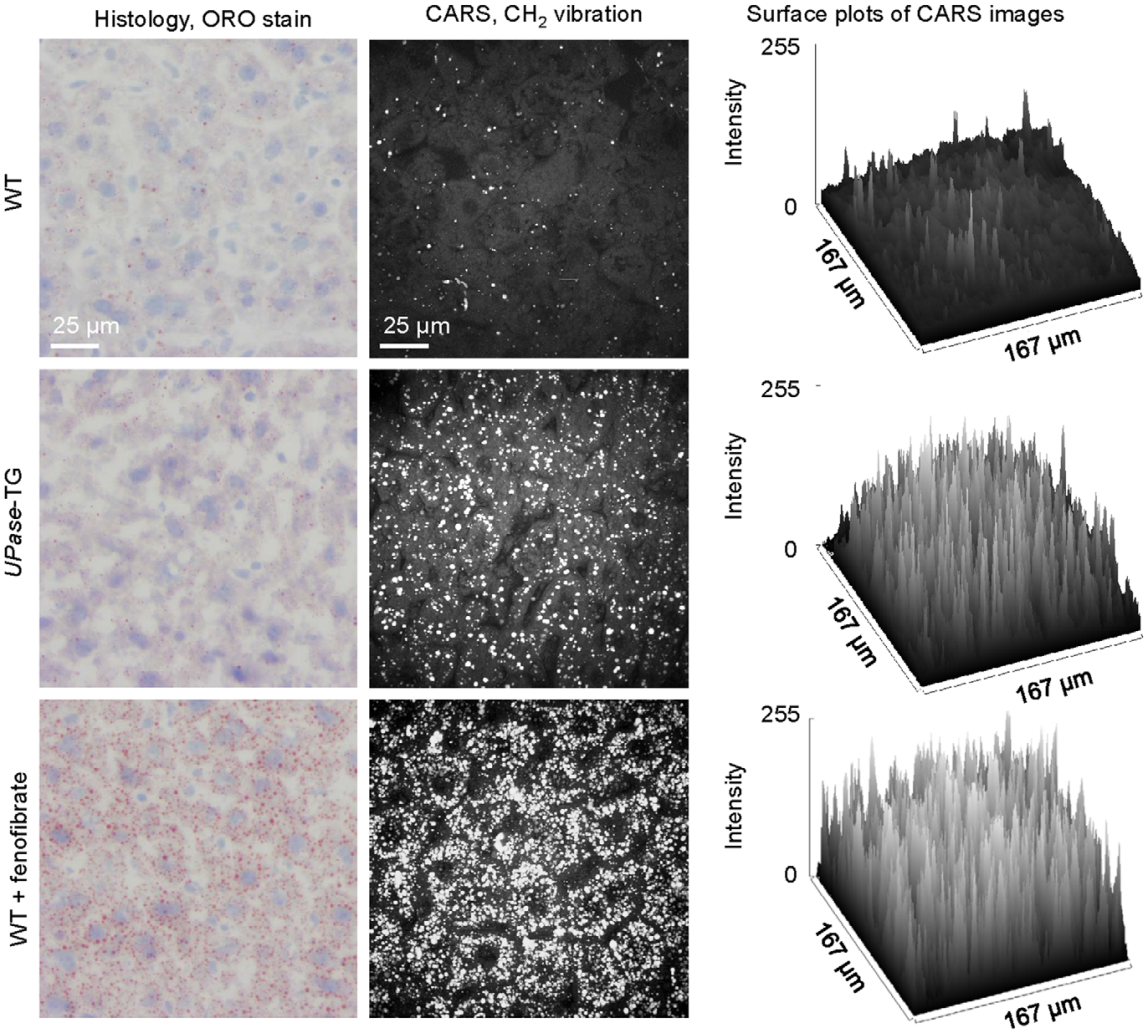
Detection of hepatic microvesicular steatosis with ORO histology and CARS microscopy. Representative ORO histology images (left column), CARS images (middle column), and surface plots of CARS images (right column) for wildtype mice with no steatosis (upper row), UPase-TG mice with mild microvesicular steatosis (middle row), and wildtype mice fed with 400 mg/kg/day fenofibrate for 5 days with severe microvesicular steatosis (lower row). Three mice, thus 3 livers, per animal group were used for direct comparison of ORO histology and CARS imaging analysis of microvesicular steatosis. Surface plots were performed with ImageJ software.

We first evaluated the ability to detect hepatic microvesicular steatosis by ORO histology and CARS microscopy. Three mice groups were employed with varying degree of hepatic microvesicular steatosis: a wildtype (WT) mice group with no microvesicular steatosis, a transgenic mice group with constitutive overexpression of uridine phosphorylase (*UPase*-TG) that exhibited mild microvesicular steatosis, and a wildtype mice group fed with 400 mg/kg/day of fenofibrate for 5 days (WT+fenofibrate) that exhibited severe microvesicular steatosis. ORO histology of liver tissues revealed no clear difference in lipid staining pattern between wildtype and *UPase*-TG mice (**Figure 1**). On the other hand, strong ORO staining of the liver tissues of mice fed with fenofibrate was detected. Our data indicates that ORO histology was sensitive for the detection of severe but not mild microvesicular steatosis. Many previous independent observations also reported on the inadequacy of ORO histology for the detection of microvesicular steatosis [16], [18].

In contrast, CARS microscopy examination of unprocessed and unstained liver tissues was highly sensitive to all types of intracellular lipid accumulation (**Figure 1**). Lipid droplets are typically strong producers of CARS signal due to high-density packing of the lipid chains. Other cellular structures that have phospholipid membrane are also producers of CARS signal although at levels significantly lower than those emitting from lipid droplets. Therefore, typical CARS images of steatosis always show strong contrasts for lipid droplets against cellular membrane backgrounds. An advantage to CARS imaging is its three-dimensional optical sectioning capability with sub-micrometric axial resolution. This 3-D imaging capability permits volumetric imaging where entire hepatocytes with 15–20 micron in thickness can be evaluated. Hence, CARS microscopy is highly-sensitive to microvesicular steatosis where the diameters of lipid droplets are in the range of 1 to several microns. On the other hand, ORO histology analysis of 5-micron thick tissue sections generally permits sampling only part of hepatocytes. Thus, ORO histology is not sufficiently sensitive for the detection of microvesicular steatosis. Our imaging data revealed an advantage of CARS microscopy over ORO histology on the detection sensitivity of hepatic microvesicular steatosis (**Figure 1**).

**Figure 2:**
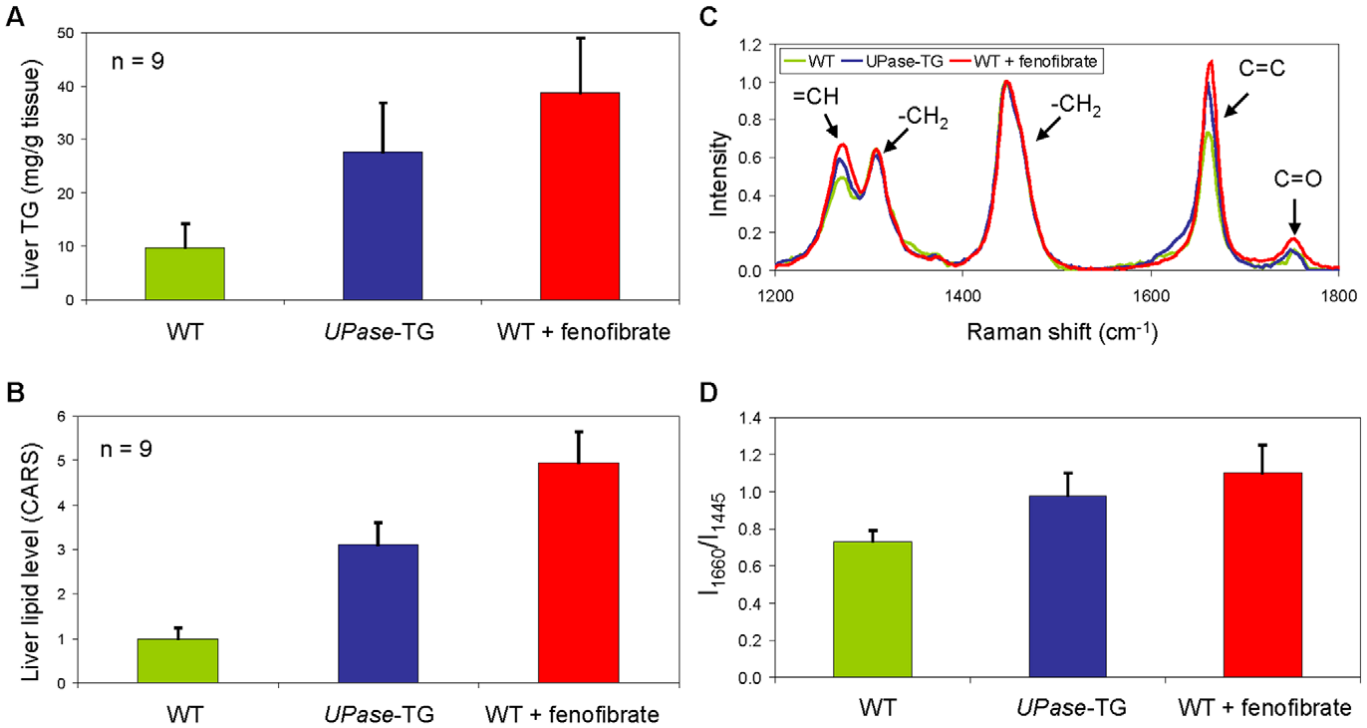
Quantitative analysis of liver lipid level and composition with biochemical assay and multimodal CARS microscopy. (**A**) Triglyceride concentration in milligram per gram liver tissue measured with a commercial triglyceride quantitation kit. (**B**) Liver lipid level determined with CARS signal intensity and normalized to 1 for wildtype mice and respectively for other animal groups. Error bars in **A** and **B** are standard deviations across 9 mice analyzed per animal group. (**C**) Representative Raman signatures of single liver lipid droplets of wildtype (light green), UPase-TG (blue), and wildtype+fenofibrate mice (red). (**D**) Lipid unsaturation (I_1660_/I_1445_) of liver lipid droplets as a function of animal group. Error bars are standard deviations across 60 lipid droplets analyzed per animal group (9 mice per group).

We next compared the quantitative capability for liver lipid content of CARS microscopy with standard biochemical assays. A standard triglyceride quantification kit was employed to measure triglyceride level in total lipid extracts of liver tissues. Triglyceride was quantified indirectly by enzymatic assays that detect glycerol from triglyceride hydrolysis [27]. Per gram of liver tissue, the triglyceride levels were measured at 10 milligrams for wildtype mice, 28 milligrams for *UPase*-TG mice, and 39 milligrams for wildtype mice fed with fenofibrate (**Figure 2A**). CARS image analysis for lipid content based on integrated CARS intensity has been described independently by many labs and found to be a reliable means for lipid content quantification [20], [28]–[30]. Using the described quantitation methods, the liver lipid level determined with CARS microscopy was normalized to 1 for wildtype mice and respectively for other mice groups. On average, the liver lipid content was 3.1 and 5 folds higher than wildtype mice for *UPase*-TG mice and wildtype mice fed with fenofibrate, respectively (**Figure 2B**). Quantitative analysis with CARS microscopy and standard biochemical assays provided different measurement units. Nonetheless, both methods concurred on the degree of steatosis in the liver tissues of three different mice groups (**Figure 2A, B**). Triglyceride quantification is a multi-step procedure prone to errors with accuracy and precision highly dependent on the quality of the reagents and enzymes and the skills of the end users [**27**]. Whereas, CARS microscopy provides direct quantification of liver lipid in a single image acquisition procedure of unprocessed and unstained tissue samples followed by software-assisted analysis. In addition to the measurement simplicity, CARS microscopy provides visual information on spatial distribution of lipid in the liver tissues, which cannot be achieved with biochemical measurements of total lipid extracts. Thus, CARS microscopy is a more simple and informative technique to evaluate the degree of liver steatosis than standard biochemical measurements.

Furthermore, the integrated spontaneous Raman microspectrometer on a CARS microscope permitted composition analysis of the liver lipid droplets in the three mice groups. Lipid droplets of all mice groups exhibited C = O carbonyl stretching vibration at 1742 cm^−1^ which is indicative of esterified fatty acids or triglyceride (**Figure 2C**) [31]. Both of the = CH deformation band at 1265 cm^−1^ and the C = C stretching band at 1654 cm^−1^ were lowest in wildtype mice, intermediate in *UPase*-TG mice, and highest in wildtype mice fed with fenofibrate. Correspondingly, the average I_1660_/I_1445_ ratio, which is a measure of lipid-chain unsaturation [32], was 0.73 for wildtype mice, 0.98 for *UPase*-TG mice, and 1.1 for wildtype mice fed with fenofibrate (**Figure 2D**). The lipid-chain unsaturation of liver lipid droplets appeared to correlate positively with the degree of hepatic microvesicular steatosis in the three mice groups examined.

**Figure 3:**
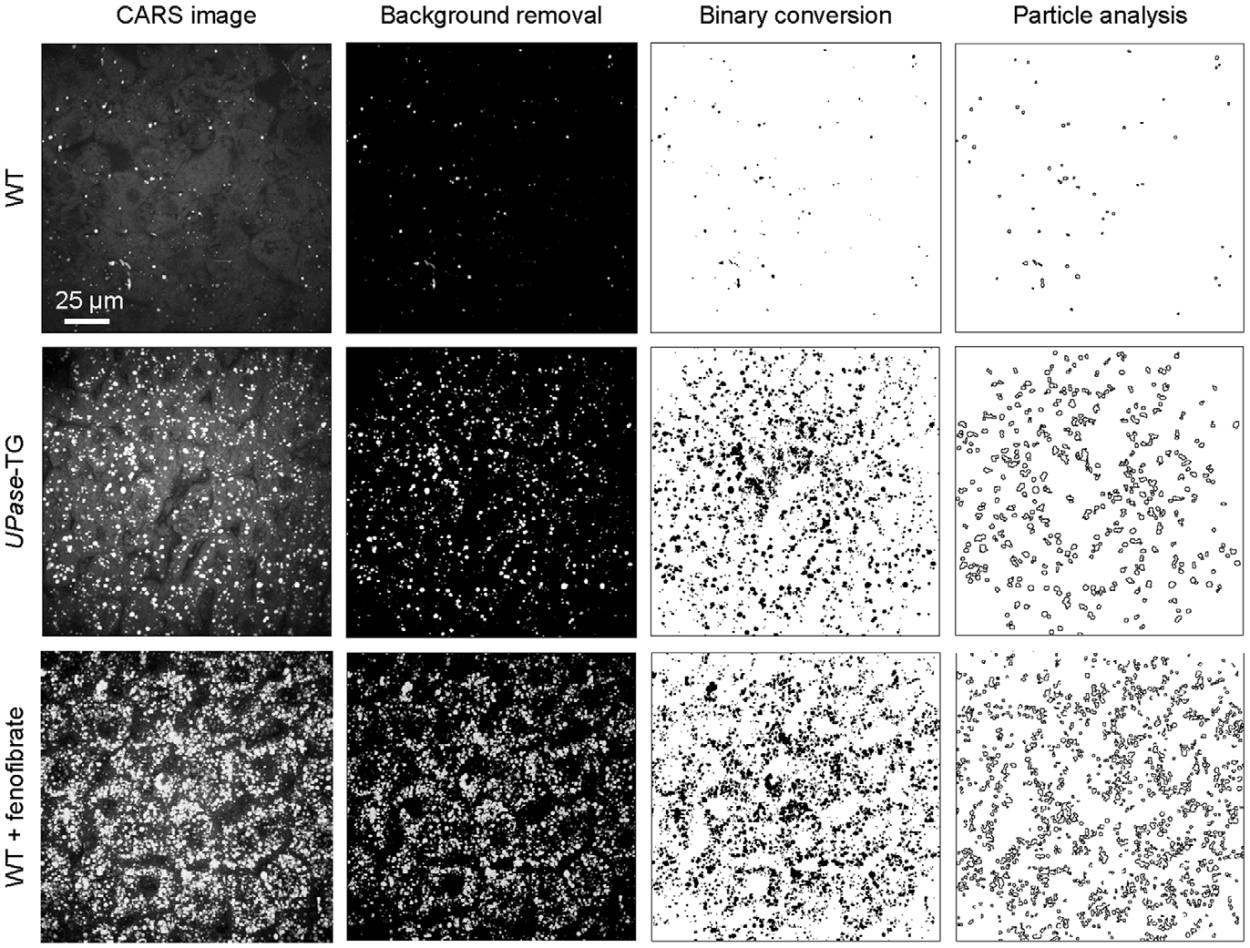
ImageJ-assisted quantitative analysis of hepatic microvesicular steatosis imaged with CARS microscopy. Columns (from left to right) describe the analysis sequence of a routine ImageJ-assisted analysis.

**Figure 4:**
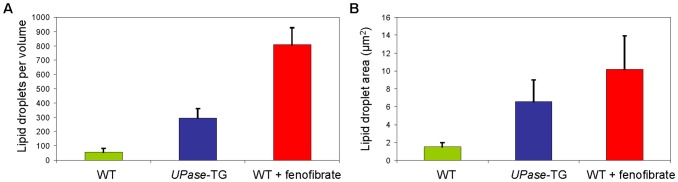
Liver lipid droplet number and size of mice groups. Liver lipid droplet number (**A**) and size (area) (**B**) as a function of mice groups determined with ImageJ-assisted analysis. Error bars are standard deviations of 9 liver volumes analyzed per mouse and 9 mice analyzed per animal group, or 81 volumes analyzed per animal group. The xyz dimensions of each analysis volume are 167 µm x 167 µm x 25 µm.

Previously, the ImageJ software, a freeware developed by the National Institutes of Health, has been applied for the analysis of lipid droplet size and number in the nematode *Caenorhabditis elegans* [30]. Here, we employed a similar automated process for the quantitation of liver lipid droplets (**Figure 3**). The lower and upper detection limits for automated lipid droplet identification were set at 0.4 micron and 5 microns to reflect the diffraction-limited optical resolution of CARS microscopy and the maximum diameter of lipid droplets in liver tissues analyzed, respectively. We found that automated enumeration and size determination of lipid droplets in wildtype and *UPase*-TG mice were fairly accurate due to the well-separation of individual lipid droplets. However, in wildtype mice fed with fenofibrate, high density of lipid droplets posed a challenge to the distinction of individual lipid droplets. Consequently, many groups of lipid droplets were rejected from the calculation due to the upper detection limit setting. Hence, automatic enumeration of lipid droplet number in severe microvesicular steatosis tissues was generally undercounted. Nonetheless, the difference between software-assisted automated enumeration and manual enumeration of lipid droplet was found to be less than 10% for all liver tissues examined (data not shown). Using ImageJ-assisted quantitation of lipid droplets, we found that per analysis volume of 167 µm x 167 µm x 20 µm, there were 50, 300, and 800 lipid droplets in wildtype mice, *UPase*-TG mice, and wildtype mice fed with fenofibrate, respectively (**Figure 4A**). On average, the areas of the lipid droplets were 1.5 µm^2^, 6.6 µm^2^, and 10.2 µm^2^ in wildtype mice, *UPase*-TG mice, and wildtype mice fed with fenofibrate, respectively (**Figure 4B**). Given the convenience and the need to eliminate human errors, computer-assisted analysis of microvesicular steatosis presents an attractive option for standardized clinical evaluation [19].

**Figure 5:**
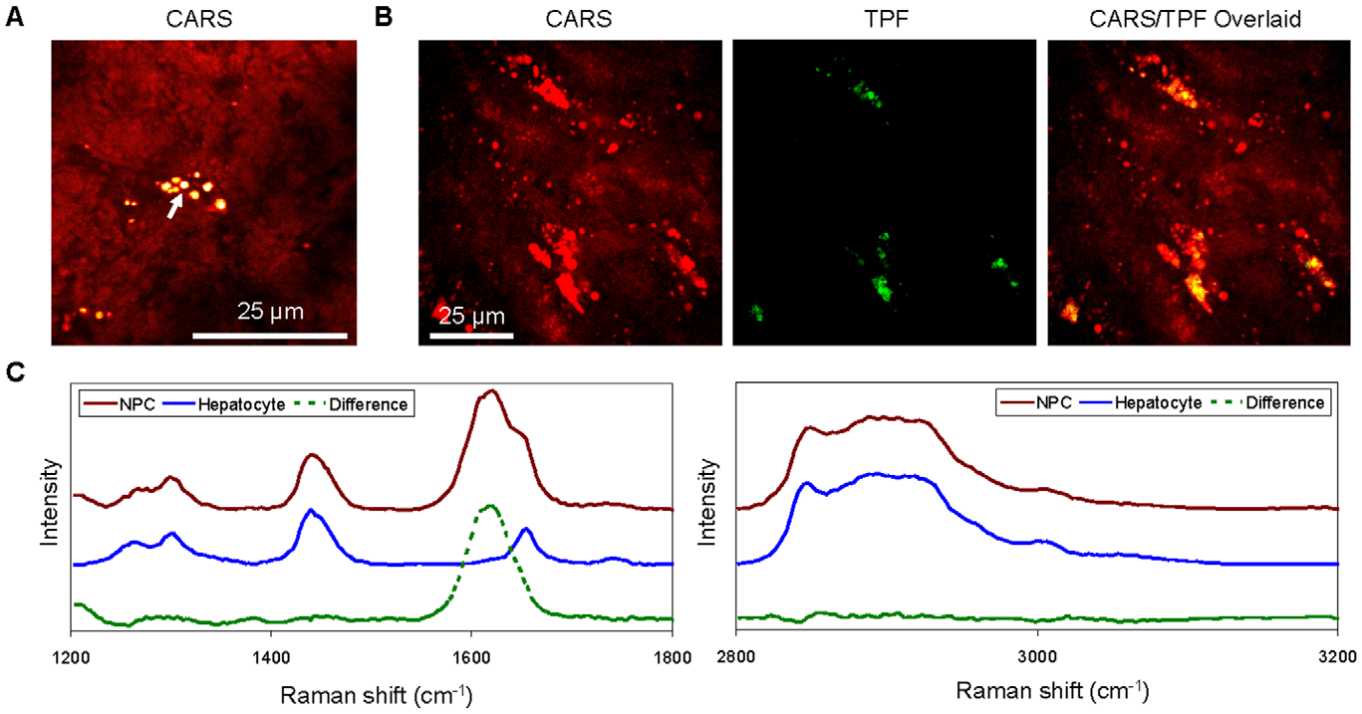
Visualization and characterization of lipid-rich non-parenchymal cells (NPCs) with CARS and TPF imaging and spontaneous Raman microspectrometry analysis. (A) CARS imaging of a lipid-rich NPC (arrow) in the interstitial space of hepatocytes. (B) CARS and TPF imaging of lipid-rich and autofluorescent lipid-rich NPCs. (C) Comparison of representative Raman spectra of hepatocytes (blue) and lipid-rich NPCs (red). Note the distinctive Raman peak at 1620 cm^−1^ for lipid-rich NPCs.

In the liver tissue of wildtype mice, we detected lipid-rich non-parenchymal cells (NPCs) in the interstitial space of hepatocytes (**Figure 5A**). Previous studies have identified these cells as Kupffer cells or resident macrophages of the liver [33]–[36]. In addition to exhibiting strong CARS signal, the lipid-rich NPCs had strong two-photon laser-induced autofluorescence detectable through a 510/42 nm emission filter (**Figure 5B**). We have previously observed autofluorescence of the same wavelengths for macrophages of atherosclerotic plaques [37] and visceral adipose tissues (VAT) [38]. We hypothesized the source of autofluorescence was from the accumulation of oxidized low-density lipoproteins and the expression of inducible nitric oxide synthase (iNOS) [37], [38]. It is plausible that strong autofluorescence in the 490 nm to 530 nm range could serve as a biomarker for label-free detection of activated tissue resident macrophages.

Raman signatures of the lipid droplets of hepatocytes and lipid-rich NPCs further revealed additional distinctive features among these cell types. Raman signatures of Kupffer cells exhibited a strong and distinctive peak that centered around 1620 cm^−1^, which was absent in hepatocytes (**Figure 5C, left panel**). It is currently unclear the origin of this Raman peak. However, activated macrophages generally have elevated expression of iNOS and its five co-factors including flavin adenine dinucleotide (FAD) and flavin mononucleotide (FMN) [39]. Both FAD and FMN have isoalloxazine rings which exhibit a Raman peak around 1620 cm^−1^ [40], [41]. Raman spectra of endothelial nitric oxide synthase (eNOS) also exhibited a prominent peak around 1620 cm^−1^ [42]. The proteins iNOS and eNOS are isoenzymes that catalyze the production of nitric oxide from L-arginine. They both share the requirement of five co-factors for enzymatic activity [39]. Hence, it is possible that this distinctive Raman peaks at 1620 cm^−1^ could be attributed to the overexpression of iNOS and its co-factors in lipid-rich NPCs.

Previously, the lipid compositions of macrophages in VAT and in atherosclerotic plaques have been examined [38], [43]. For analysis of macrophages of atherosclerotic plaques, the Raman spectral window examined was between 2750 cm^−1^ and 3050 cm^−1^ [43]. Hence, no information can be extracted regarding the 1620 cm^−1^ peak. For analysis of VAT macrophages, the entire Raman spectral window from 800 cm^−1^ to 3100 cm^−1^ was presented [38]. However, the 1620 cm^−1^ peak was not detected despite elevated expression of iNOS in VAT macrophages. A key difference between VAT macrophages and lipid-rich NPCs was the cell shapes. While VAT macrophages exhibited normal cell shapes, lipid-rich NPCs exhibited highly distorted cell shapes due to their location in the tight interstitial spaces of hepatocytes. The compact cell shapes of lipid-rich NPCs rendered their cytoplasmic volumes much smaller than those of VAT macrophages or macrophages of atherosclerotic plaques. The axial resolution at full-width-at-half-maximum of the probed volume of spontaneous Raman microspectrometry was determined to be approximately 6.4 µm [31]; whereas, lipid droplets of lipid-rich NPCs generally exhibited diameters of less than 5 µm. Thus, the probed volumes for spontaneous Raman microspectrometry analysis were larger than those of lipid droplets. It is well-observed that iNOS is a cytoplasmic protein with no reported association with lipid droplets [39]. Therefore, it is likely that the Raman signatures of lipid droplets of lipid-rich NPCs had the contribution of cytoplasmic iNOS.

In addition, the Raman peak intensities at 2850 cm^−1^ and 2935 cm^−1^, which represent CH_2_ and CH_3_ symmetric stretching bands, respectively, were identical in both hepatocytes and lipid-rich NPCs (**Figure 5C, right panel**). The I_2850_/I_2935_ ratio is a measure of lipid packing order, which is a function of temperature or lipid-chain unsaturation [31], [32]. Our data suggest identical lipid-chain unsaturation and lipid packing order for lipid droplets of both hepatocytes and lipid-rich NPCs.

**Figure 6:**
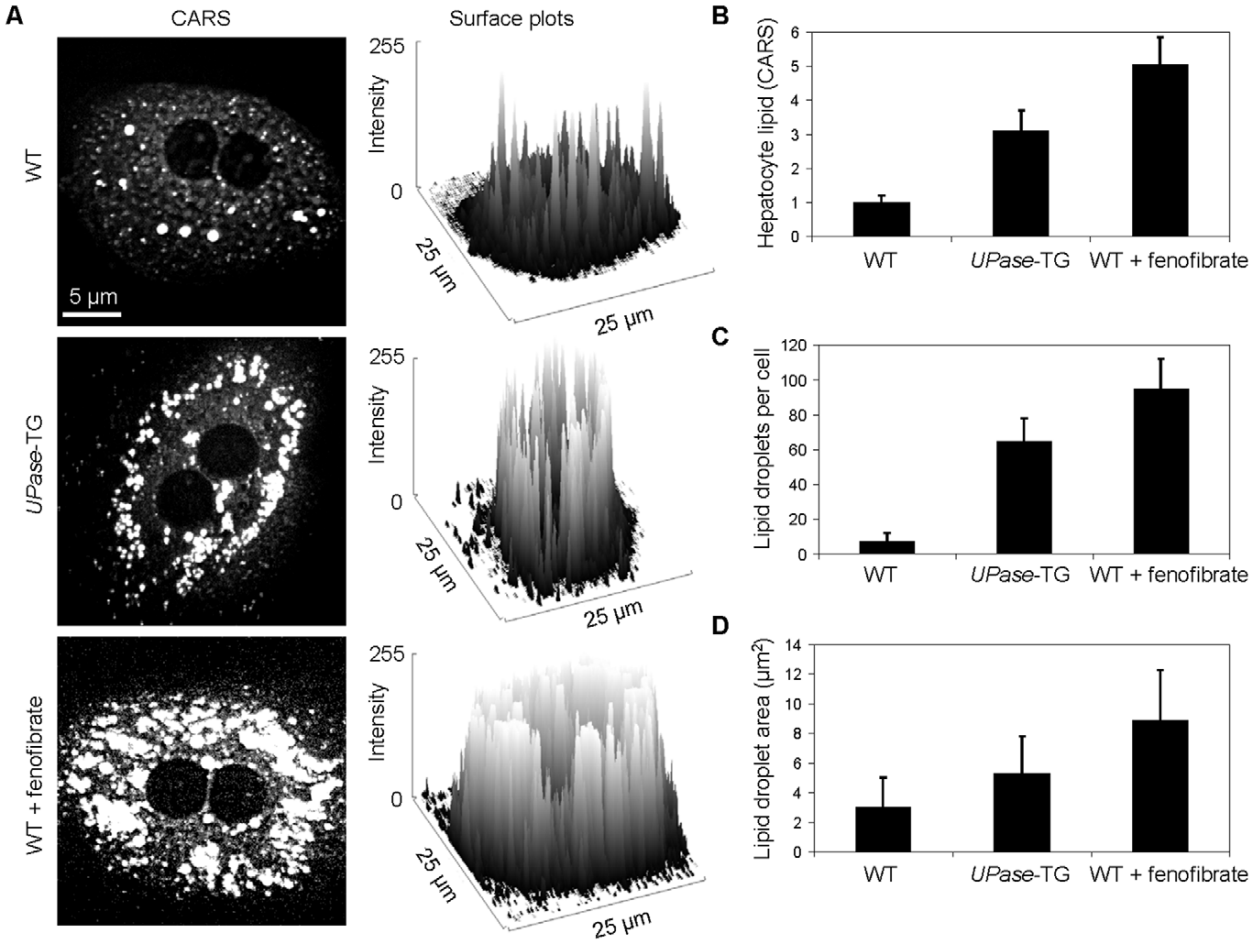
Primary hepatocyte cultures for *in vitro* studies. (**A**) CARS images and surface plots of representative primary hepatocytes at 24 hours post-isolation. Quantitative analysis performed on CARS images for (**B**) lipid level within individual hepatocyte, (**C**) lipid droplets per hepatocyte, and (**D**) lipid droplet areas. Error bars are standard deviations across 60 hepatocytes evaluated per animal group.

A unique advantage of CARS microscopy is its non-perturbative imaging capability that permits visualization of living systems [44]. To explore this capability for the studies of microvesicular steatosis, we applied CARS microscopy toward imaging primary hepatocyte cultures. Using a previously described isolation method and growing condition [45], we were successful at isolating and culturing primary hepatocytes from the three mice groups. We found that primary hepatocytes preserved their phenotype at 24 hours after isolation (**Figure S2**). However, consistent with many previous reports [46], de-differentiation of hepatocytes was observed at 48 hours after isolation. At 96 hours after isolation, cultured primary hepatocytes acquired spindle-like shapes with membrane protrusion into tentacle-like structures. Loss of phenotype might be accompanied with loss of hepatic function [46]. Therefore, we only evaluated cultured primary hepatocytes at up to 32 hours post isolation. We found that cultured primary hepatocytes from the three animal groups generally reproduced their *in vivo* lipid phenotypes (**Figure 6A–D**).

**Figure 7:**
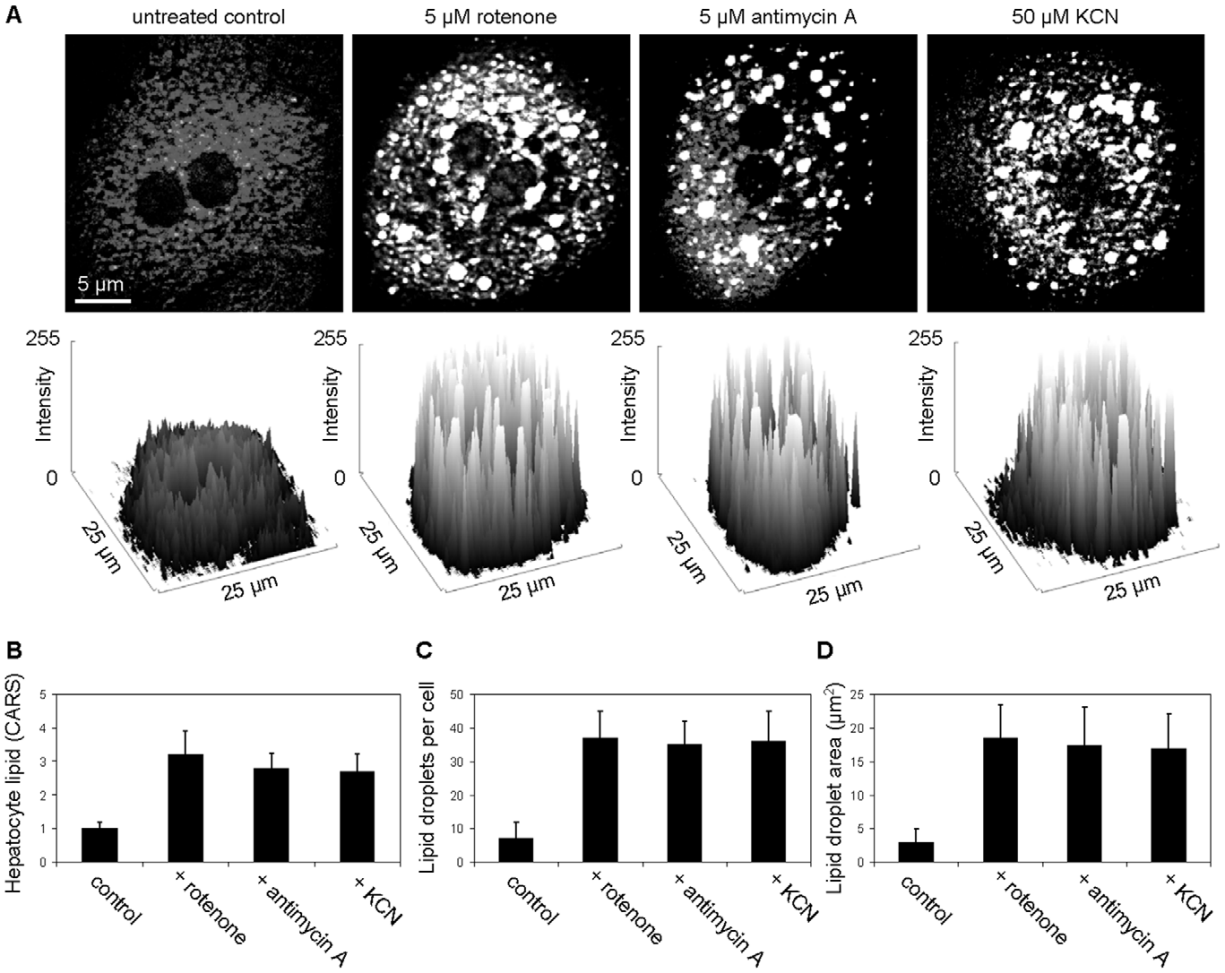
Mitochondrial toxins induced microvesicular steatosis in cultured primary hepatocytes. (**A**) CARS images and surface plots of representative hepatocytes treated with mitochondrial toxins for 24 hours. Quantitative analysis performed on CARS images for (**B**) lipid level within individual hepatocyte, (**C**) lipid droplets per hepatocyte, and (**D**) lipid droplet areas. Error bars are standard deviations across 60 hepatocytes evaluated per animal group.

Primary cultures of hepatocytes have been widely used for the evaluation of drugs on hepatotoxicity [47]. Among the causes of drug-induced hepatic microvesicular steatosis is the impairment of the mitochondrial function [48], [49]. Hence, we applied CARS microscopy to examine the impacts of mitochondrial toxins on the lipid phenotype of cultured hepatocytes. We found that exposure to known mitochondria chemical toxins such as rotenone, antimycin A, and potassium cyanide caused microvesicular steatosis in cultured hepatocytes (**Figure 7A–D**). Thus, microvesicular steatosis could be a potential indirect read-out of mitochondrial impairment, which can be detected with high sensitivity using CARS microscopy.

## Discussion

We showed that CARS microscopy provided a simple and versatile means for the characterization of hepatic microvesicular steatosis. For histopathology analysis, CARS microscopy was more sensitive in detecting hepatic microvesicular steatosis compared to ORO histology. For the evaluation of liver lipid content, quantitation of CARS intensity agreed with biochemical triglyceride measurement. Liver tissues did not have to be processed or stained for CARS imaging analysis, thus, eliminating sample preparation time, cost of dyes, enzymes, and reagents, and most importantly errors associated with traditional methods. Integrated spontaneous Raman microspectroscopy permitted analysis of lipid-unsaturation of lipid droplets. Furthermore, computer-assisted analysis permitted quantitation of lipid droplet size and number, which should complement the steatosis analysis with CARS intensity measurement. CARS microscopy provided informative and quantitative analysis of hepatic microvesicular steatosis in a single experimental procedure. With its measurement sensitivity, simplicity, and robustness, CARS microscopy should be an invaluable tool for the clinical evaluation of hepatic microvesicular steatosis.

The multimodal imaging capability of a standard CARS microscope provides a powerful means for pathological analysis of liver tissues. We have shown in this paper the integrative use of CARS, two-photon fluorescence (TPF), and spontaneous Raman microspectroscopy to identify lipid-rich NPCs. This capability should permit evaluation of liver tissue inflammation through the detection and enumeration of lipid-rich NPCs. Other researchers have previously employed CARS and SHG to evaluate hepatic macrovesicular steatosis simultaneously with fibrosis [21], [22]. Thus, a multimodal nonlinear optical microscope configured for simultaneous CARS, SHG, and TPF imaging together with spontaneous Raman microspectroscopy analysis should permit evaluation of various stages of fatty liver disease. Fortunately, such multimodality can be achieved on any standard CARS microscope [26], [50], [51]. The versatility of CARS microscopy should render it an indispensable tool for the studies of fatty liver disease.

Label-free and non-perturbative imaging capabilities are unique advantages of CARS microscopy that permit visualization of living systems. Indeed, CARS microscopy has been applied to visualize intestinal lipid absorption [52] and central and peripheral nervous systems [53]–[55] in living mice. However, *in vivo* application of CARS microscopy is still faced with many challenges namely the limited penetration depth of optical microscopy, the image distortion due to respiration motion, and the infancy of CARS endoscopy development [24]. On the other hand, CARS microscopy should find robust applications on *in vitro* live tissues and cell culture systems [56], [57]. Specifically for mammalian cell culture systems, CARS microscopy has been widely applied for the visualization of intracellular lipid droplet formation, mobilization, and remodeling [58]–[62]. Here, we presented an example on the use of CARS microscopy to screen for microvesicular steatosis due to the exposure of primary hepatocytes to mitochondrial toxins. We anticipate CARS microscopy as a rapid detection means for *in vitro* evaluation of the effects of drugs on hepatotoxicity.

A potential drawback of CARS microscopy for the imaging of biological sample is the existence of a non-resonant background [23], [24]. This CARS non-resonant background is Raman-shift independent. However, it can cause distortion to CARS spectral peaks and make the comparison between CARS spectra and spontaneous Raman spectra difficult, especially in the congested spectral regions. The interference of this non-resonant background is significant when CARS microscopy is applied to image structures with low concentration of molecular vibrator, or those with Raman signatures in the congested fingerprint region between 800 cm^−1^ to 1800 cm^−1^. On the other hand, CARS resonant signal is Raman-shift dependent and is quadratically dependent on the concentration of the molecular vibrator. When CARS microscopy is applied to visualize lipid-rich structures, which have high density of carbon-hydrogen (C-H) vibrational oscillator, the resonant signal at the C-H stretch vibrational frequency around 2851 cm^−1^ is much larger than non-resonant signal. Large resonant CARS signal for lipid-rich structures permits selective visualization of lipid droplets and myelin sheaths with high resonant-to-non-resonant ratio [26]. Shown in this paper and in many publications from other CARS research groups [20], [28], [43], [44], [63]–[65], non-resonant contribution from water or any other non-lipid biological structures constitutes a relatively low background at 2851 cm^−1^, which does not interfere with the visualization of lipid-rich structures. In addition, such low non-resonant background can also be removed through subtraction of an image taken at a non-C-H vibrational frequency from an image taken at a C-H vibrational frequency around 2851 cm^−1^ [66]. Alternatively, any CARS microscope can be modified into a stimulated Raman scattering (SRS) microscope, which is a background-free vibrational imaging technique, with the addition of a laser intensity modulator, a photodiode detector, and a lock-in amplifier [67].

Another challenge to clinical application of CARS microscopy is the standardization of image presentation and data analysis. CARS signal intensity level, which is commonly used as an indicator of CH_2_ and lipid concentration, is a variable function dependent on the laser source, laser power, spatial overlap and temporal synchronization of probe and pump lasers, laser-scanning rate, quality of optical lens and detectors, mode of detection (epi-reflected versus forward detection), and a broad range of other factors. It is likely that CARS signal intensity level of the same tissue sample will vary significantly with different CARS microscope systems; thus, will hinder the comparison of CARS images in a future clinical image depository. To address this challenge, we present CARS images together with 3-D surface plots, where background and signal levels for every pixel are shown. 3-D surface plots provide accurate information on the performance of the CARS microscope system, where the signal-to-noise ratios can be compared among CARS images of similar tissue samples. In addition, we present data on lipid droplet number and size in addition to CARS image intensity for the evaluation of hepatic microvesicular steatosis. Lipid droplet number and size are parameters that are unlikely to be varied due to different CARS microscope systems or the training level of end users. Currently, we extracted quantitative information from CARS image using the open-access NIH ImageJ software. The accuracy of our quantitation is constraint by the capability of the ImageJ software. Fortunately, improved CARS image segmentation and quantitative algorithms are being developed [68], [69]. Future standardization of CARS image presentation and data mining will facilitate widespread clinical adoption of CARS microscopy.

The adoption of CARS microscopy for clinical applications needs to overcome several additional challenges. First, the footprint of a typical CARS microscope is still quite large and can occupy an entire standard procedure room. Second, the operation of CARS microscopy requires certain level of training in advanced optics. Third, the cost of CARS microscopy is quite prohibitive mainly due to the cost of the laser sources [24]. And fourth, the small field-of-view typical of any single CARS image limits the tissue areas that can be surveyed. Fortunately, the use of fiber light sources described recently should help to reduce both the footprint and cost of CARS microscopy [70]–[73]. The commercial versions of CARS microscope introduced in recent years are also making operation of CARS microscopy more user-friendly. In addition, large-area surface mapping through the use of image stitching hardware and software has overcome the sampling area problem and permitted visualization of entire rodent brains and cross-section of blood vessels [54], [74], [75]. With many recent technical advances, it is foreseeable that CARS microscopy will become a standard tool for pathologists in the near future.

## Materials and Methods

### Animals

Three mice groups were used in our experiments, C57BL/6 mice or wildtype mice (Jackson Laboratories, Bar Harbor, ME), *UPase-*TG mice with C57BL/6 background, and C57BL/6 mice fed with 400 mg/kg/day of fenofibrate for 5 days. The generation and characterization of *UPase*-TG mice that constitutively over-express uridine phosphorylase 1 are being described in detail in a separate paper. All mice used were male of 10–12 weeks old. All mice were fed with PicoLab Mouse Diet 20 ground pellets (Cat. No. 5058, LabDiet, Brentwood, MO). For the mice group receiving fenofibrate feeding, fenofibrate was thoroughly mixed with ground pellets. Fenofibrate-induced mitochondrial impairment, hepatotoxicity, and fatty liver in rodents have been previously described [76]–[78]. All animal studies were performed with the approval of the Desert Research Institute Animal Care and Use Committee.

### A Multimodal CARS Microscope

The basic setup of the CARS microscope system has been described previously [38]. Briefly, the signal (924.2 nm) and idler (1255 nm) outputs of an optical parametric oscillator (OPO, Levante Emerald, Berlin, Germany) were used as the pump and Stokes beams, respectively, to produce a frequency difference of 2851 cm^−1^. The laser sources were attenuated with neutral density filters, passed through a laser scanner (C1plus, Nikon, Melville, NY) and focused with a 60× IR objective into the sample with a combined power of 66 mW. The same laser sources were used for simultaneous CARS and TPF imaging. Epi-reflected signal was directed into a multi-channel detector, spectrally separated with dichroic mirrors, selected with bandpass filters (Semrock, Lake Forest, IL), and detected with red-sensitive photomultiplier tubes (R10699, Hamamatsu, Japan). Bandpass filters for TPF autofluorescence signal and CARS signal were 510/42 nm and 736/128 nm, respectively. Images were acquired at approximately 1 second per frame. Volumetric imaging of liver tissues were performed by taking approximately 26 frames taken along the vertical axis at 1-micron intervals to produce 3D volumes of 167 µm x 167 µm x 25 µm in xyz dimensions. Generally, 9 volumes were obtained per liver tissue examined. For display purpose, stacked 3D images were presented.

### Computer-assisted Quantitative Analysis of CARS Images

For quantitative analysis of liver lipid content, average CARS pixel intensity for 26 frames was obtained for each volume using the ImageJ software. Background pixel intensity, which includes nonresonant signal and signal arising from cellular membrane, was determined and subtracted from average CARS pixel intensity. Square-root of the corrected CARS pixel intensity yields a numerical value for liver lipid content. Liver lipid content was normalized to 1 for wildtype mice and correspondingly for *UPase*-TG mice and wildtype mice fed with fenofibrate. Analysis of CARS signal intensity as a measure of lipid level has also been described previously in the literature [20], [28]–[30]. The area and number of lipid droplets were measured using the particle sizing function provided by the ImageJ software on 3-D stacked images as described previously [30]. The liver tissues of 9 mice per animal group were evaluated with CARS microscopy. On average, 9 liver volumes were analyzed per mouse and 9 mice analyzed per animal group, or 81 volumes analyzed per animal group. The xyz dimensions of each analysis volume were 167 µm x 167 µm x 25 µm.

### Spontaneous Raman Microspectroscopy

A continuous-wave laser at 671 nm (Laserglow, Toronto, Ontario, Canada) was attenuated to 20 mW and collinearly combined with CARS laser beams to serve as the excitation light source for Raman microspectroscopy. A spectrometer (Shamrock SR-303i-A, Andor Technology, Belfast, U.K.) was mounted to the backport of the microscope (Ti-E, Nikon, Melville, NY). Confocal Raman was achieved by selecting scattered light with a bandpass filter and focusing it with a 100-mm focal length achromatic lens into a 50-micron pinhole. The spectrometer was equipped with a 300 grooves/mm 500-nm blaze angle grating and a thermoelectrically cooled back-illuminated electron-multiplying charge-coupled device (EMCCD; Newton DU970N-BV, Andor Technology, Belfast, U.K.). CARS laser sources were blocked prior to Raman microspectrometry measurement. Raman data were collected by averaging three 4-second acquisitions. On average, Raman microspectrometry analysis was performed on 60 hepatocyte lipid droplets per animal group. Raman microspectrometry analysis was performed on lipid droplets of approximately 10 non-parenchymal cells per liver tissue of 9 wildtype mice.

### Liver Sample Preparation

Liver was perfused with phosphate buffered saline prior to collection. Collected liver tissues of the same mice were divided into halves with one half used for standard histopathology analysis or biochemical assays and the other half used for CARS imaging. For CARS imaging, liver tissues were sliced with an oscillating tissue slicer (EMS 4500, Electron Microscopy Sciences, Hatfield, PA) into 200-micron thick sections. Liver tissue sections were transferred into glass-bottom chambered slides and examined with CARS microscopy.

### Analysis of Liver Lipid with Histology and Biochemical Assays

Hepatic microvesicular steatosis was assessed with standard ORO histology by a histopathology lab on 3 mice per animal group. Typical thickness of a histology section is approximately 5 microns. Hepatic triglyceride level was determined using the Triglyceride Quantification Kit (Cat. No. 10010303, Cayman Chemical, Ann Arbor, MI) and normalized with liver tissue weight. Hepatic triglyceride level was measured for 9 mice per animal group.

### Primary Hepatocyte Isolation and Culturing

Mouse hepatocytes were isolated using a two-step collagenase perfusion technique initially described by Seglen [45]. Culture medium consisted of DMEM/F12 supplemented with 10% FBS, 20 ng/mL epidermal growth factor, 40 ng/mL dexamethasone, 1X insulin/transferrin/selenium supplement, and MycoZap Plus-PR antibiotic mixture. Viability ranged from 70–80% as determined by trypan blue exclusion. Hepatocyte enrichment at 4 hours after plating reached approximately 90–95%. Hepatocytes were plated in collagen coated glass-bottom culture dish (Cat. No. P35GCOL-1.5-14-C, MatTek Corporation, Ashland, MA). For the evaluation of the lipid phenotypes of primary hepatocytes, 3 mice per animal group were used for primary hepatocytes collection and 3 culture dishes of primary hepatocytes per mouse were used for the evaluation of lipid phenotypes. For the evaluation of mitochondrial toxins on hepatocyte lipid phenotypes, 3 wildtype mice were used for the collection of primary hepatocytes and 3 culture dishes of primary hepatocytes per mouse were used for evaluation. Primary hepatocytes were treated with 5 µM rotenone, 5 µM antimycin A, or 50 µM KCN for 24 hours at 37°C and 5% CO_2_ prior to examination with CARS microscopy. Culture dishes with plated hepatocytes were removed from incubator and placed directly onto the CARS microscopy for imaging at ambient temperature. Quantitative analyses were performed on approximately 60 primary hepatocytes per animal group. Primary hepatocytes were generally used for treatment at 4 to 8 hours post-isolation. All hepatocytes, untreated and treated, were evaluated before 32 hours post-isolation.

## Supporting Information

Figure S1 **Non-specific staining of liver tissues by Oil Red O.** (**A**) CARS (grey) and two-photon fluorescence (TPF, red) imaging of a liver tissue stained with Oil Red O. ORO exhibits laser-induced fluorescence detectable through a 570/40 nm emission filter. (**B**) Bright field image of the same liver tissue section. The liver tissue belongs to a 1 year old C57BL/6 mouse that has been placed on a high fat diet regimen for 6 months. Liver tissue was sliced into 200-micron thick section, stained with ORO for 15 minutes, and washed thoroughly 6 times over a 6 hours period to removed unstained ORO prior to imaging. This liver tissue exhibits macrovesicular steatosis detectable with CARS microscopy.
(TIF)

Figure S2 **De-differentiation of purified and plated primary hepatocytes of wildtype mice.** Primary hepatocytes acquire spindle-like shapes at 96 hours post-isolation. Images were taken with phase contrast microscopy.
(TIF)
