# Supplementary figures and images for: Label-free Evaluation of Hepatic Microvesicular Steatosis with Multimodal Coherent Anti-Stokes Raman Scattering Microscopy
Source: PLoS One. 2012 Nov 30;7(11):e51092. doi: 10.1371/journal.pone.0051092 (PMC3511365; doi:10.1371/journal.pone.0051092)

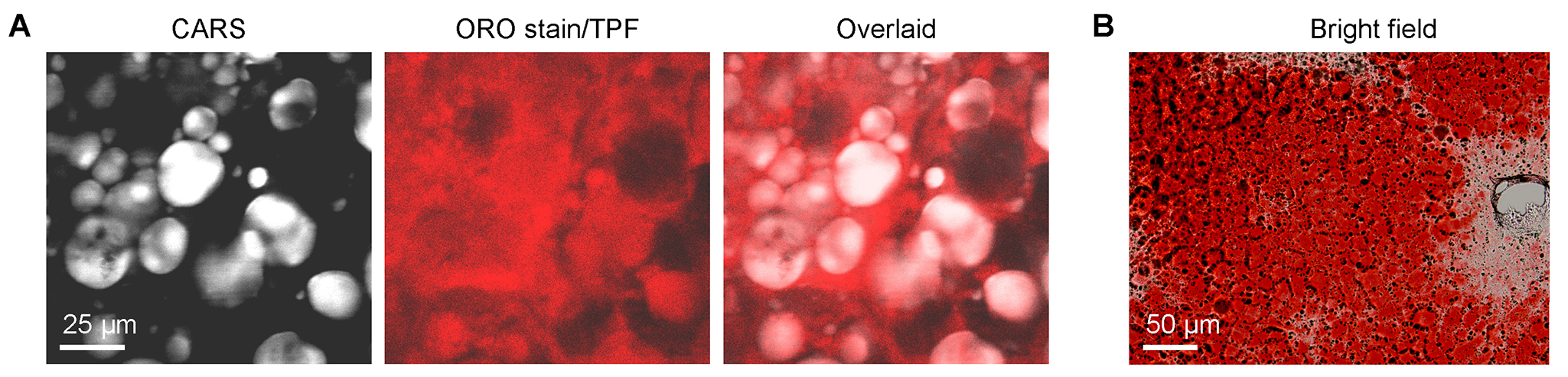

Supplement: Figure S1 — Non-specific staining of liver tissues by Oil Red O. (A) CARS (grey) and two-photon fluorescence (TPF, red) imaging of a liver tissue stained with Oil Red O. ORO exhibits laser-induced fluorescence detectable through a 570/40 nm emission filter. (B) Bright field image of the same liver tissue section. The liver tissue belongs to a 1 year old C57BL/6 mouse that has been placed on a high fat diet regimen for 6 months. Liver tissue was sliced into 200-micron thick section, stained with ORO for 15 minutes, and washed thoroughly 6 times over a 6 hours period to removed unstained ORO prior to imaging. This liver tissue exhibits macrovesicular steatosis detectable with CARS microscopy. (TIF) [file pone.0051092.s001.tif]

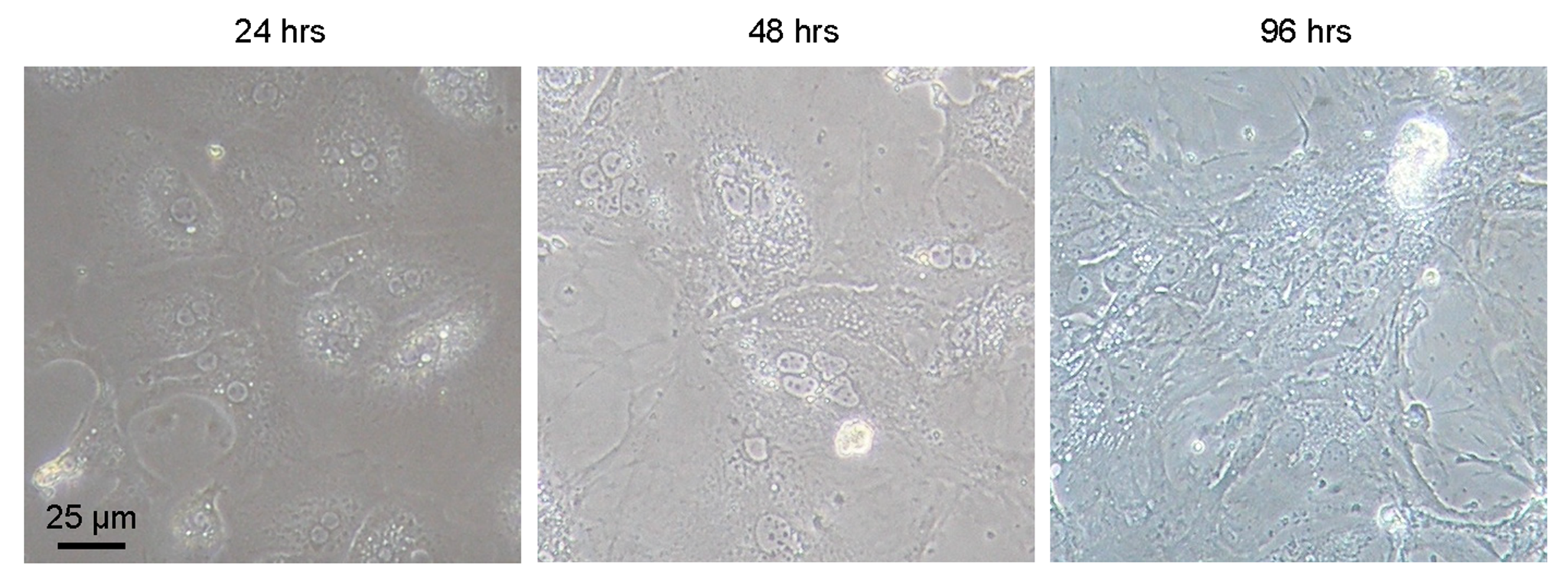

Supplement: Figure S2 — De-differentiation of purified and plated primary hepatocytes of wildtype mice. Primary hepatocytes acquire spindle-like shapes at 96 hours post-isolation. Images were taken with phase contrast microscopy. (TIF) [file pone.0051092.s002.tif]
